# mRBioM: An Algorithm for the Identification of Potential mRNA Biomarkers From Complete Transcriptomic Profiles of Gastric Adenocarcinoma

**DOI:** 10.3389/fgene.2021.679612

**Published:** 2021-07-27

**Authors:** Changlong Dong, Nini Rao, Wenju Du, Fenglin Gao, Xiaoqin Lv, Guangbin Wang, Junpeng Zhang

**Affiliations:** ^1^Center for Informational Biology, School of Life Sciences and Technology, University of Electronic Science and Technology of China, Chengdu, China; ^2^School of Life Sciences and Technology, University of Electronic Science and Technology of China, Chengdu, China; ^3^Key Laboratory for NeuroInformation of the Ministry of Education, University of Electronic Science and Technology of China, Chengdu, China

**Keywords:** complete transcriptomic profiles, biomarkers, sample classification, prognosis, generalization ability

## Abstract

**Purpose:**

In this work, an algorithm named mRBioM was developed for the identification of potential mRNA biomarkers (PmBs) from complete transcriptomic RNA profiles of gastric adenocarcinoma (GA).

**Methods:**

mRBioM initially extracts differentially expressed (DE) RNAs (mRNAs, miRNAs, and lncRNAs). Next, mRBioM calculates the total information amount of each DE mRNA based on the coexpression network, including three types of RNAs and the protein-protein interaction network encoded by DE mRNAs. Finally, PmBs were identified according to the variation trend of total information amount of all DE mRNAs. Four PmB-based classifiers without learning and with learning were designed to discriminate the sample types to confirm the reliability of PmBs identified by mRBioM. PmB-based survival analysis was performed. Finally, three other cancer datasets were used to confirm the generalization ability of mRBioM.

**Results:**

mRBioM identified 55 PmBs (41 upregulated and 14 downregulated) related to GA. The list included thirteen PmBs that have been verified as biomarkers or potential therapeutic targets of gastric cancer, and some PmBs were newly identified. Most PmBs were primarily enriched in the pathways closely related to the occurrence and development of gastric cancer. Cancer-related factors without learning achieved sensitivity, specificity, and accuracy of 0.90, 1, and 0.90, respectively, in the classification of the GA and control samples. Average accuracy, sensitivity, and specificity of the three classifiers with machine learning ranged within 0.94–0.98, 0.94–0.97, and 0.97–1, respectively. The prognostic risk score model constructed by 4 PmBs was able to correctly and significantly (^∗∗∗^*p* < 0.001) classify 269 GA patients into the high-risk (*n* = 134) and low-risk (*n* = 135) groups. GA equivalent classification performance was achieved using the complete transcriptomic RNA profiles of colon adenocarcinoma, lung adenocarcinoma, and hepatocellular carcinoma using PmBs identified by mRBioM.

**Conclusions:**

GA-related PmBs have high specificity and sensitivity and strong prognostic risk prediction. MRBioM has also good generalization. These PmBs may have good application prospects for early diagnosis of GA and may help to elucidate the mechanism governing the occurrence and development of GA. Additionally, mRBioM is expected to be applied for the identification of other cancer-related biomarkers.

## Introduction

Gastric cancer is a global health problem, with more than 1 million patients being diagnosed worldwide each year. Gastric cancer remains the third leading cause of cancer-related death, despite a worldwide decline in morbidity and mortality over the past 5 years (Bray et al., 2018; Thrift and El-Serag, 2020). Gastric adenocarcinoma (GA) is a type of gastric cancer caused by malignant transformation of gastric gland cells. Incidence of GA accounts for approximately 95% of gastric malignancies (Lawrence, 2004), and GA pathogenesis has not been fully elucidated. Five-year survival rate of early gastric cancer can reach >90% (Tan, 2019), and 5-year survival rate of patients with advanced gastric cancer is only 20–40% (Siegel et al., 2016; Song Z. et al., 2017). Therefore, an improvement in early diagnosis and treatment of GA can decrease GA incidence and mortality.

Several studies have suggested that molecular biomarkers are important for early diagnosis, treatment, and evaluation of prognosis of cancer (Parker et al., 2009; Collins and Varmus, 2015; Pellegrini et al., 2015). According to the central dogma of biology, RNA carries genetic and regulatory information that reflects the state of the cells. RNA biomarkers have considerably higher sensitivity and specificity for the detection of cancer samples compared with those of protein biomarkers and can more dynamically reflect cellular states and regulatory processes to provide additional cellular information compared with that provided by DNA biomarkers (Xi et al., 2017). Furthermore, miRNAs can regulate gene expression by binding to mRNAs or related proteins (Bartel, 2009). LncRNAs can competitively bind miRNAs as competing endogenous RNAs (ceRNAs) to regulate gene expression and cellular functions (Xia et al., 2014; Song Y. X. et al., 2017). Therefore, mRNAs occupy a key position in the complex regulatory processes involving three types of biomolecules. Abnormal expression of mRNAs in the key positions of the regulatory network can easily bias the overall stability of the network. mRNAs may cause abnormal activation of one or more signaling pathways, which also leads to abnormal expression or function of the biomolecules in these signaling pathways to promote physiological and tissue disorders, such as cancer (Lu et al., 2016; Duan et al., 2020; Hu et al., 2020; Wei et al., 2020). mRNAs that occupy the key positions are more likely to be biomarkers.

Many mRNA biomarkers associated with occurrence and development of GA were identified using experimental and computational methods. Representative studies can be summarized as follows. Yoon et al. (2019) confirmed that the activation of KRAS in GA cells stimulates epithelial-to-mesenchymal transition to form cancer stem-like cells, thereby promoting metastasis. Huang C. et al. (2020) found that overexpression of DGKi in GA indicates poor prognosis, and the MAPK signaling pathway may be one of the key pathways that regulate occurrence and development of GA by DGKi. Necula et al. (2020) showed that overexpression of COL10A1 in GA patients is associated with poor survival and that COL10A1 can be used as a potential biomarker for early detection of GA. Wang (2017) identified 446 differentially expressed (DE) mRNAs in the gene expression profile related to gastric cancer, used these DE mRNAs to construct a protein-protein interaction network, and finally identified five key mRNAs in the protein-protein interaction network (COL5A2, TOP2A, KIF20A, FN1, and PRC1). However, existing GA-related mRNA biomarkers are not sufficient to provide accurate GA diagnosis in the clinic and thoroughly elucidate GA pathogenesis. Identification of GA-related mRNA markers with high sensitivity and specificity is of great significance for early diagnosis, targeted therapy, and analysis of prognosis of GA. Therefore, this study first proposes an algorithm to identify potential mRNA biomarkers (PmBs) related to GA based on complete transcriptomic RNA (including mRNA, lncRNA, and miRNA) profiles of GA. The proposed algorithm evaluates the potential of an mRNA with abnormal expression as GA biomarker in the regulation of transcriptional coexpression and at the protein-protein interaction level. The integrated analysis of multiple omics data objectively avoids the problems of signal noise and high inaccuracy caused by single omics analysis. Then, the sample classification power and prognostic relevance of PmBs were analyzed to assess their reliability and value for assistance with clinical diagnosis. The novelty of this paper are as follows:

1. An novel algorithm named mRBioM for the identification of potential mRNA biomarkers from complete transcriptomic profiles of GA was developed.
2. A cancer-related factor was proposed to distinguish whether a single sample is cancer or normal, which may have good application prospects in the personalized diagnosis of cancers.
3. The mRBioM-based prognostic risk score model was constructed to assess the overall survival rate of cancer patients.

## Materials and Methods

### Data Collection

The complete transcriptome TCGA-STAD dataset of RNAs (including mRNA, lncRNA, and microRNA) of GA patients published by various countries was obtained from the Genomic Data Commons of National Cancer Institute in July, 2019. The pathological tissue types of the source data were limited to GA. The dataset included 279 GA patients and the corresponding clinical information (Table 1). The dataset included 257 cases that had only GA tissue samples, 20 cases that had GA and paired paracancerous tissue samples, and 2 cases that had only paracancerous tissue samples. Detailed information about these 299 samples is shown in Supplementary Table 1.

**TABLE 1:** Statistics of clinical information of included 279 GA patients.

**Clinical variables**		**Number of sample (n)**	**n% (%)**
Gender	Male	171	61.3
	Female	108	38.7
Age	<40	2	0.7
	40–60	86	30.8
	60–80	174	62.4
	=80	17	6.1
Oncology classification	Adenocarcinoma, intestinal type	45	16.1
	Adenocarcinoma, NOS	119	42.7
	Adenocarcinoma, diffuse type	55	19.7
	Papillary adenocarcinoma, NOS	5	1.8
	Tubular adenocarcinoma	55	19.7
Pathological staging	Stage I	33	11.8
	Stage II	94	33.7
	Stage III	118	42.3
	others	34	12.2

*Stage I includes I, IA, and IB, Stage II includes II A and II B, and Stage III includes III A, III B, and III C; NOS, Not Otherwise Specified.*

**FIGURE 1:**
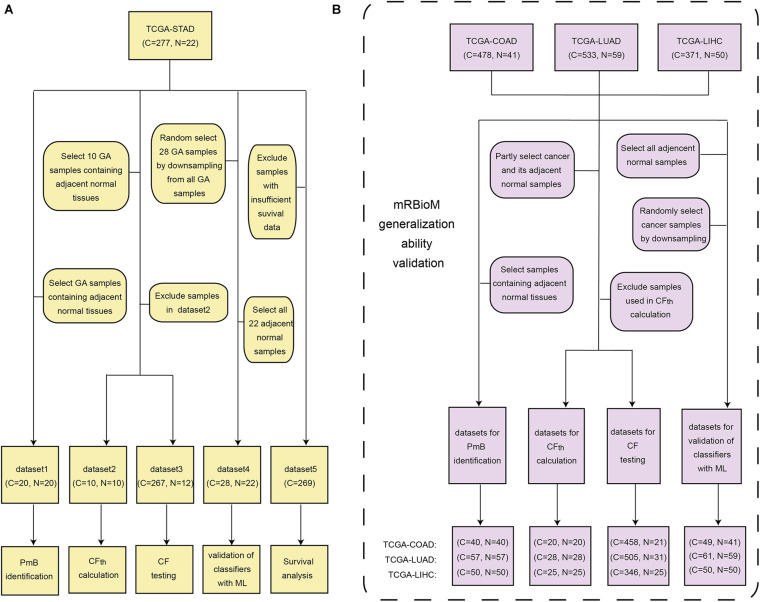
Data organization and utilization. **(A)** Five subsets from the TCGA-STAD dataset. **(B)** TCGA-COAD, TCGA-LUAD, and TCGA-LIHC. C, cancer sample; N, adjacent normal sample; CF, cancer-related factor; CF_th_, threshold of CF; ML, machine learning.

TCGA-STAD was organized into five subsets for various studies: dataset 1 for GA-related PmB identification, datasets 2–4 for evaluation of PmB classification, and dataset 5 for survival analysis, as shown in Figure 1A. Three other cancer-related RNA transcriptomic profiles were downloaded from the Genomic Data Commons database in May of 2020 and were used to verify the generalization ability of mRBioM: TCGA-COAD, including 478 cases of colon cancer and 41 cases of normal tissues; TCGA-LUAD, including 533 cases of lung adenocarcinoma and 59 cases of normal tissues; and TCGA-LIHC, including 371 cases of liver cancer and 50 cases of normal tissues. The characteristics of the three datasets are shown Figure 1B.

### mRBioM Algorithm

The amount of information for a molecule can determine whether this molecule is in a key position in the regulatory network (Teschendorff et al., 2014). Thus, mRBioM identified PmBs by evaluating the amount of information for each DE mRNA based on the transcriptional coexpression relationships between DE mRNAs, miRNAs, and lncRNAs and in the PPI network. The steps of the mRBioM algorithm are described below.

#### DE RNA Analysis

The *limma* package of R (Ritchie et al., 2015) was used to identify DE RNAs from dataset 1 containing 20 GA and 20 paracancer samples (a total of 40 samples) from TCGA-STAD. Dataset 1 was preprocessed by cleaning and standardization; next, the logarithm of the expression fold change (FC) of each RNA in GA vs. adjacent normal samples was calculated. The log_2_FC value and corresponding corrected *p*-value (represented by Padj) of each RNA were used to determine whether an RNA was differentially expressed. The screening conditions for DE RNAs in this study were Padj < 0. 05 or 0.01 and |*log*_2_FC | 1.

#### Calculation of the Coexpression Correlation Coefficient Matrix for RNAs

Suppose that we identified *N*, *J*, and *K* DE mRNAs, DE miRNAs, and DE lncRNAs, respectively. The expression vector of each DE RNA in all samples was extracted from dataset 1. Pearson correlation coefficients *M*_xy_ and *L*_xz_ between DE mRNA *x*(*x* = 1,⋅, *N*) and DE miRNA *y*(*y* = 1,⋅, *J*) and between DE mRNA *x* and DE lncRNA *z*(*z* = 1,⋅, *K*), respectively, were calculated according to Eqs. (1) and (2).

(1) $$M_{x\,y}=\frac{\sum_{i=1}^{40}\left(x_{i}-\bar{x}\right)\,\left(y_{i}-\bar{y}\right)}{\sqrt{\sum_{i=1}^{40}{\left(x_{i}-\bar{x}\right)}^{2}\,\sum_{i=1}^{40}{\left(y_{i}-\bar{y}\right)}^{2}}}$$

(2) $$L_{x\,z}=\frac{\sum_{i=1}^{40}\left(x_{i}-\bar{x}\right)\,\left(z_{i}-\bar{z}\right)}{\sqrt{\sum_{i=1}^{40}{\left(x_{i}-\bar{x}\right)}^{2}\,\sum_{i=1}^{40}{\left(z_{i}-\bar{z}\right)}^{2}}}$$

where *x_i_, y_i_*, and *z*_i_ and $\bar{x}$, $\bar{y}$, and $\bar{z}$ are the *i-th* element and the average value of all elements in the expression vectors of DE mRNA *x*, DE miRNA *y*, and DE lncRNA *z*, respectively. Pearson correlation coefficients between all DE mRNAs and DE miRNAs and between all DE mRNAs and DE lncRNAs constitute two correlation coefficient matrixes, which are represented by **M** (*N* × *J*) and **L** (*N* × *K*), respectively.

#### Calculation of the Amount of Information for DE mRNA in the Coexpression Network

The connection of each molecule in the regulatory network is influenced by many factors, such as environment and diet, and has a degree of uncertainty that accounts for the amount of information for each molecule (Teschendorff et al., 2014). In this study, we propose to use the information rate of a DE mRNA in the transcriptional coexpression networks to measure the uncertainty of its connection and then use Shannon’s information entropy theory to estimate the amount of coexpression information for a DE mRNA.

The information rate for DE mRNA *x* in the coexpression network between DE mRNA and DE miRNA was defined as the ratio of a significant pearson correlation coefficient (*p* < 0.05) in the *x-th* line corresponding to DE mRNA *x* in **M** to the sum of all significant pearson correlation coefficients (*p* < 0.05) in the *x-th* line of **M**, which measures the correlation degree between a DE mRNA *x* and a DE miRNA *y* (*y* = 1,⋅, *J’*). All information rates for DE mRNA *x* associated with other DE miRNAs constitute the information rate vector **p_x_** defined by Eq. (3). Similarly, the information rate vector **q_x_** for DE mRNA *x* in the coexpression network of DE mRNAs and DE lncRNAs is defined according to Eq. (4).

(3) $${\text{p}}_{x}=\frac{{\text{M}}_{x}^{\prime }}{\sum_{y=1}^{J^{\prime }}M_{x\,y}^{\prime }}$$

(4) $${\text{q}}_{x}=\frac{{\text{L}}_{x}^{\prime }}{\sum_{z=1}^{K^{\prime }}L_{x\,z}^{\prime }}$$

where ${\text{M}}_{\text{x}}^{{}^{\prime \prime }}$nd **L***’***_x_** are the vectors composed of the pearson correlation coefficients with statistical *p* values less than 0.05 in the *x-th* row of **M** and **L**, respectively; *M’*_xy_ (*y* = 1, 2,⋅, *J’*) and *L’*_xz_ (*z* = 1, 2, ⋅, *K’*) are the pearson correlation coefficients with statistical *p-*values less than 0.05 in the *x-th* row of **M** and **L**, respectively; and *J’* and *K’* are the corresponding numbers.

According to Shannon’s information entropy theory, the amount of coexpression information for DE mRNA *x* (expressed as *S*_RNA_*_x_*) is estimated by Eq. (5).

(5) $$S_{R\,N\,A\,x}=\sum_{y=1}^{J^{\prime }}-p_{x\,y}\,l\,o\,g_{2}\,p_{x\,y}+\sum_{z=1}^{K^{\prime }}-q_{x\,z}\,l\,o\,g_{2}\,q_{x\,z}$$

where *p*_xy_ is the *y-th* information rate in **p_x_**, *y* = 1, 2, ⋅,*J’*; *q*_xz_ is the *z-th* information rate in **q_x_**, *z* = 1, 2,⋅, *K’*.

#### Estimation of the Amount of Information for DE mRNA in the Protein-Protein Interaction Network

We constructed a protein-protein interaction network based on the protein interaction information of all DE mRNAs acquired from the online STRING database^1^. Higher protein-protein connectivity score in the protein-protein interaction network corresponds to greater amount of interaction information between two proteins (Szklarczyk et al., 2019). Therefore, we used cs to measure the amount of protein interaction information (represented by *S*_PPI_*_x_*) that corresponds to DE mRNA *x* according to Eq. (6).

(6) $$S_{P\,P\,I\,x}=1+\sum_{j\in N}c\,s_{x\,j}$$

where *cs*_xj_ = 1) represents the connection score between a protein encoded by DE mRNA *x* and a protein encoded by another DE mRNA *j* (*j*∋*N*, *j≠x*).

#### Identification of PmBs Associated With GA

The sum of *S*_RNA_*_x_* and *S*_PPI_*_x_* normalized by maximum was used as the total information amount of DE mRNA *x* (denoted by *S*_x_) according to Eq. (7).

(7) $$S_{x}=\frac{S_{R\,N\,A\,x}}{m\,a\,x\,\left\{S_{R\,N\,A\,x}\right\}}+\frac{S_{P\,P\,I\,x}}{m\,a\,x\,\left\{S_{P\,P\,I\,x}\right\}}$$

All DE mRNAs were sorted according to *S*_x_ (*x* = 1, 2 ⋅, *N*), and PmBs were identified based on the change trend of *S*_x_ (*x* = 1, 2⋅,*N*). The number of identified PmBs was recorded as Q. Gene Ontology (GO) enrichment and Kyoto Encyclopedia of Genes and Genomes (KEGG) pathway analyses of PmBs were performed by the *clusterProfiler* R package to investigate the functions of PmBs (Yu et al., 2012).

### Evaluation of Sample Classification Power of PmBs

We designed four classifiers based on PmBs to discriminate the positive GA and negative control samples to illustrate the value of PmBs identified by mRBioM in auxiliary clinical diagnosis. The performance of the four classifiers was evaluated by sensitivity, specificity, and accuracy.

#### Cancer-Related Factor

The cancer-related factor of a sample was determined by the expression values of PmBs in the samples and was used to discriminate the sample types. The cancer-related factor value of a sample was defined as the ratio of the average logarithm values of the expression of upregulated and downregulated PmBs in the sample according to Eq. (8).

(8) $$C\,F=\frac{\frac{1}{n_{u\,p}}\times \sum_{i=1}^{n_{u\,p}}\log_{2}E\,x_{u\,p}\,\left(i\right)}{\frac{1}{n_{d\,n}}\times \sum_{j=1}^{n_{d\,n}}\log_{2}E\,x_{d\,n}\,\left(j\right)}$$

where *CF* indicates the value of cancer-related factor. *n*_up_nd *Ex*_up_(*i*) are the number of upregulated PmBs and the expression value of the *i-th* upregulated PmB in a sample, respectively. Similarly, *n*_dn_nd *Ex*_dn_(*j*) are the number of downregulated PmBs and the expression value of the *j-th* downregulated PmB.

We randomly selected *n* (in this instance, *n* = 10) GA and adjacent normal samples from the mRNA expression profile of dataset 1 to identify the best *CF* threshold for discrimination of the positive and negative samples, and only the expression value of *Q* PmBs from each sample was used to form dataset 2 (*C* = 10, *N* = 10). Next, the expression profiles of 2*n* samples in dataset 2 were converted into a new expression profile containing *n* samples. The expression value vector **S_m_** (dimension is 1 × *n*) of the *m-*th (*m* = 1, 2,⋅,*Q*) PmB in the synthetic expression profile was calculated according to Eq. (9).

(9) $$S_{m}=\frac{S_{\mathrm{tm}}+S_{\mathrm{nm}}\times \frac{\sum_{i=1}^{n}S_{t\,m}\,\left(i\right)}{\sum_{i=1}^{n}S_{n\,m}\,\left(i\right)}}{2}$$

where **S_tm_** (dimension is 1 × *n*) and **S_nm_** (dimension is 1 × *n*) are the expression value vectors of the GA and control samples in dataset 2 of *m-th* (*m* = 1, 2,⋅,*Q*) PmB, respectively. *S*_tm_(*i*) and *S*_nm_(*i*) are the *i-th* expression value elements (*I* = 1, 2,⋅,*n*) of **S_tm_** (dimension is 1 × *n*) and **S_nm_** (dimension is 1 × *n*), respectively.

Next, Eq. (8) was used to calculate the *CF* of the *i-th* sample in the generated expression profile (denoted as *CF*_i_, *i* = 1, 2,⋅,*n*), and the geometric mean value of the *CF* values of *n* samples (Eq. 10) was used as the threshold of *CF* (denoted as *CF*_th_).

(10) $$C\,F_{t\,h}=\sqrt[{n}]{\prod_{i=1}^{n}C\,F_{i}}$$

Finally, the samples of dataset 2 were excluded from TCGA-STAD, and the remaining samples only with the expression values of *Q* PmB were used to form dataset 3 (*C* = 267, *N* = 12), which was used to test the ability of cancer-related factor to recognize the GA samples. If the *CF* of a sample was greater than *CF*_th_, the sample was identified as GA (positive); otherwise, the sample was identified as control (negative).

#### Classifiers With Machine Learning

Three classifiers with machine learning based on random forest (RF) (Wang H. et al., 2020), support vector machine (SVM) (Zhang et al., 2017; Zhang and Liu, 2017), and naive Bayes (NB) (Dou et al., 2015) were constructed using the normalized expression values of PmBs as the classification feature implemented by *randomForest* R package (Liaw and Wiener, 2002), the svm function of the *e1071* R package (Meyer et al., 2019), and the NaiveBayes function of the *klaR* R package (Weihs et al., 2005), respectively. Of course, there are other improved Bayesian models that can replace NB classification algorithms (Nagarajan et al., 2013; Thapa et al., 2018). Since the unbalanced sample size between the GA and control groups will affect the classification effect of the three classifiers, we used the downsampling method to randomly extract 28 samples from 277 GA samples and retain all 22 adjacent normal samples in TCGA-STAD, which formed validation dataset 4 (*C* = 28, *N* = 22). Finally, the performance of the three classifiers with machine learning was confirmed on dataset 4 by using the fivefold cross-validation method.

### PmBs-Based Survival Analysis

We excluded 10 patients with missing survival time or less than 30 day survival from the cohort of 279 patients in TCGA-STAD to exclude patients who died from other factors and finally used the transcription profiles of 269 GA patients with 55 PmBs to form dataset 5 for survival analysis. The average survival time of GA patients in dataset 5 was 21.575 ± 17.506 months, and 105 GA patients died at the end of follow-up, accounting for 39% of the total cohort.

Clinical information about patients (Supplementary Table 1) and dataset 5 (*C* = 269) were integrated, and a univariate Cox regression model of the *survival* R package (Peterschmitt et al., 2018) was used to identify survival-related PmBs that have a significant impact on survival time (*p* < 0.05); then, a multivariate Cox regression model was used to determine *T* survival-related PmBs to construct a prognostic risk model (Lossos et al., 2004) used to calculate the survival-based risk score of a patient (Eq. 11).

(11) $$\mathrm{Risk}\,\mathrm{score}=\sum_{t=1}^{T}E\,x\,p_{P\,m\,B}\,\left(t\right)\times W_{P\,m\,B}\,\left(t\right)$$

where *Exp*_PmB_(*t*) is the expression value of *t-th* survival-related PmB in the patient sample, and *W*_PmB_(*t*) is the corresponding multivariate Cox regression coefficient of *t-th* survival-related PmB, *t* = 1, 2,⋅, *T*.

Then, the median of the risk scores of all patients in dataset 5 was used as the cutoff value to divide the patients into the high- and low-risk groups. Finally, Kaplan–Meier analysis was used to assess the overall survival rate of patients in the high- and low-risk groups, and the log-rank test was used to determine whether there is a significant difference in the overall survival rate of patients in the high-risk vs. low-risk groups. In addition, we used the *survivalROC* package (Kamarudin et al., 2017) of R to perform ROC curve analysis to evaluate the sensitivity and specificity of the prognostic risk model.

## Results

### DE mRNAs and PmBs in GA

**FIGURE 2:**
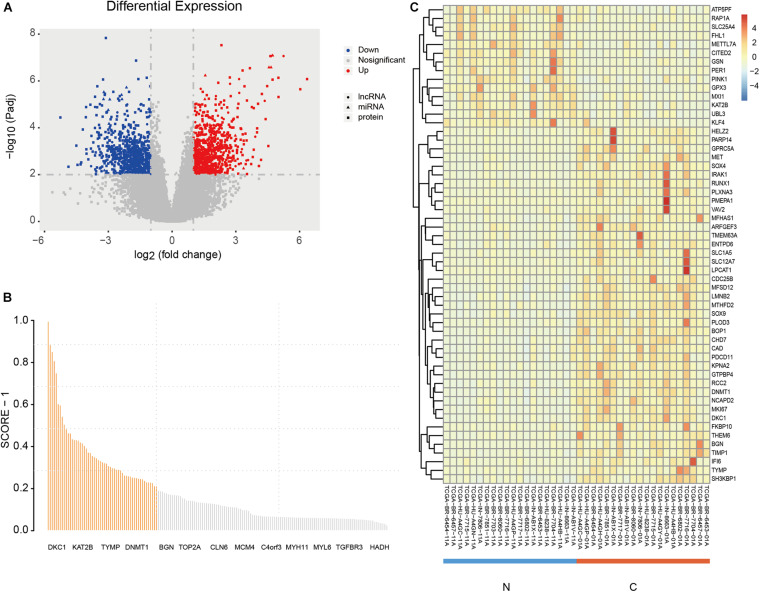
DE RNAs and the screening results of PmBs. **(A)** Volcano plot of DE RNAs (circles: DE mRNA, squares: DE miRNA, triangles: DE lncRNA); red dots represent up-regulated DE RNAs, and blue dots represent down-regulated DE RNAs. **(B)** The total information amount plot of DE mRNAs; the abscissa represents symbols of mRNA (part of the symbols is displayed), and the ordinate is the total information amount of each DE mRNA. **(C)** Heatmap of the PmBs of adjacent normal group vs. GA group. DE, differentially expressed; PmBs, potential mRNA biomarkers; N, adjacent normal sample; C, cancer sample; TIA, total amount of information.

A total of 170 DE mRNAs |*log*_2_FC(| 1, Padj < 0.01), 623 DE lncRNAs |*log*_2_FC(| 1, Padj < 0.05), and 52 DE miRNAs |*log*_2_FC(| > 1, Padj < 0.01) were obtained. Figure 2A shows the volcano plots of significantly DE RNAs, the details of all DE mRNAs are shown in Supplementary Table 2. And the results of the protein-protein interacti network analysis are shown in the attached file “string_protein_interactions_170.tsv.”

The total information amount for each DE mRNA was calculated by mRBioM, and the curve constructed by total information amount of all DE mRNAs from large to small is shown in Figure 2B. There is a significant decrease of curve after the orange area and finally the curve tends to be stable. Therefore, a total of 55 DE mRNAs with total information amount corresponding to the orange region were identified as PmBs for further study (Table 2). A literature search confirmed that 13 PmBs were related to GA (23.64%), and 27 PmBs were related to other cancers (49.09%) (Table 2). The expression distribution of 55 PmBs is shown in Figure 2C, corresponding to 41 upregulated PmBs (lower right corner vs. lower left corner) and 14 downregulated PmBs (upper right corner vs. upper left corner).

**TABLE 2:** The identified PmBs and their total information amount.

**No.**	**PmB symbol**	**TIA**	**Relevance to cancer**	**No.**	**PmB symbol**	**TIA**	**Relevance to cancer**
1	MET	1.485	GA (Ebert et al., 2019)	29	PMEPA1	1.290	PCa (Sharad et al., 2020)
2	KLF4	1.808	GA (Zhao R. et al., 2020)	30	DNMT1	1.277	BRCA (Wang et al., 2021)
3	LPCAT1	1.885	GA (Uehara et al., 2016)	31	MFHAS1	1.264	CRC (Chen et al., 2016)
4	SOX4	1.356	GA (Ding et al., 2019)	32	IRAK1	1.264	BRCA (Li Y. et al., 2020)
5	KPNA2	1.506	GA (Tsai et al., 2016)	33	TIMP1	1.418	PCa (Guccini et al., 2021)
6	GPX3	1.393	GA (Cai et al., 2019)	34	RCC2	1.255	BRCA (Chen et al., 2019)
7	TYMP	1.374	GA (Huang et al., 2016)	35	SLC12A7	1.254	AC (Brown et al., 2018)
8	FKBP10	1.430	GA (Wang R. G. et al., 2020)	36	IFI6	1.239	MM (Cheriyath et al., 2007)
9	CDC25B	1.347	GA (Kudo et al., 1997)	37	BGN	1.231	CRC (Chen et al., 2020)
10	SOX9	1.299	GA (Wang H. et al., 2020)	38	GTPBP4	1.229	LUC (Zhang et al., 2020)
11	GPRC5A	1.260	GA (Liu et al., 2016)	39	RUNX1	1.261	CRC (Li et al., 2019)
12	CITED2	1.250	GA (Gao et al., 2020)	40	MXI1	1.214	LUC (Huang et al., 2018)
13	FHL1	1.214	GA (Xu et al., 2012)	41	TMEM63A	1.751	Not reported
14	DKC1	1.996	CRC (Hou et al., 2020)	42	PDCD11	1.598	Not reported
15	PLOD3	1.464	LUC (Baek et al., 2019)	43	METTL7A	1.467	Not reported
16	KAT2B	1.543	BRCA (Zhang et al., 2017)	44	ATP5PF	1.852	Not reported
17	PARP14	1.433	MM (Barbarulo et al., 2012)	45	UBL3	1.433	Not reported
18	VAV2	1.352	BRCA (Wang P. et al., 2020)	46	HELZ2	1.405	Not reported
19	MTHFD2	1.421	RCC (Lin et al., 2018)	47	SLC25A4	1.321	Not reported
20	RAP1A	1.372	LUC (Huang N. et al., 2020)	48	ARFGEF3	1.328	Not reported
21	LMNB2	1.437	HCC (Kong et al., 2020)	49	NCAPD2	1.298	Not reported
22	PER1	1.339	LUC (Lin et al., 2020)	50	ENTPD6	1.604	Not reported
23	GSN	1.248	CRC (Kim et al., 2018)	51	CAD	1.253	Not reported
24	CHD7	1.310	EC (Lu et al., 2020)	52	THEM6	1.333	Not reported
25	SLC1A5	1.321	CRC (Ma et al., 2018)	53	MKI67	1.247	Not reported
26	PLXNA3	1.306	BRCA (Gabrovska et al., 2011)	54	PINK1	1.232	Not reported
27	BOP1	1.292	CRC (Killian et al., 2006)	55	SH3KBP1	1.232	Not reported
28	MFSD12	1.292	MM (Wei et al., 2019)				

*GA, gastric adenocarcinoma; PCa, prostate cancer; CRC, colorectal cancer; BRCA, breast cancer; AC, adrenocortical carcinoma; MM, myeloma; LUC, lung cancer; RCC, renal cell carcinoma; HCC, hepatocellular carcinoma; EC, endometrial cancer; TIA, total information amount.*

### Functional Enrichment Analysis of PmBs in GA

**FIGURE 3:**
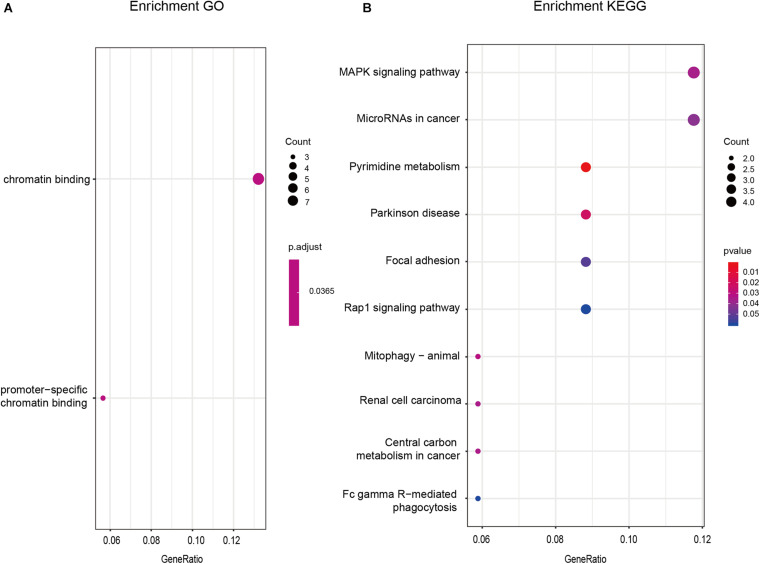
GO and KEGG enrichment analysis of PmBs. **(A)** GO enrichment analysis. **(B)** KEGG enrichment analysis. GO, Gene Ontology; KEGG, Kyoto Encyclopedia of Genes and Genomes; GA, gastric adenocarcinoma.

GO and KEGG functional enrichment analyses were performed by *clusterProfiler* of R using 55 PmBs to investigate the potential functions of these biomarkers. As shown in Figure 3A, the GO terms indicated that these 55 PmBs were mainly concentrated in chromatin binding (*p* < 0.05). The results of KEGG analysis with *p* < 0.05 suggested that these 55 PmBs were mainly related to pathways closely associated with occurrence and development of cancer, such as mitophagy-animal, ribosome biogenesis in eukaryotes, MAPK signaling pathway, cAMP signaling pathway, central carbon metabolism, microRNAs in cancer, and renal cell carcinoma (Figure 3B).

### Sample Classification Power of Cancer-Related Factor

**FIGURE 4:**
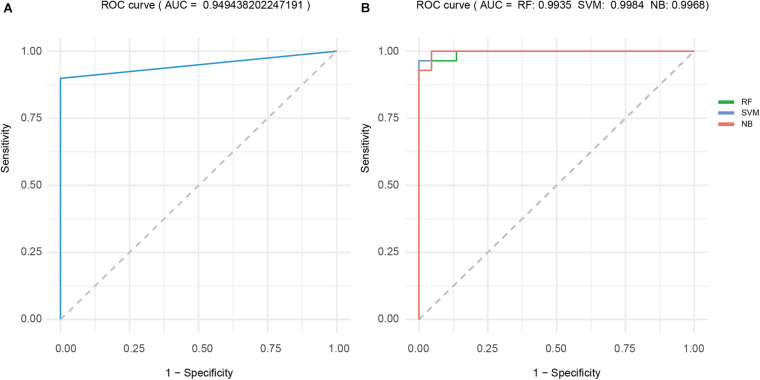
ROC curve analysis for the four classifiers. **(A)** CF. **(B)** RF-based, SVM-based and NB-based classifiers. ROC, receiver operating characteristic; CF, cancer factor; RF, random forest; SVM, support vector machine; NB, naive Bayes.

*CF*_th_ of dataset 2 was 0.9725, and the remaining samples in TCGA-STAD formed dataset 3 to test the sample classification power of cancer-related factor (Table 3). Table 3 shows that accuracy, sensitivity, and specificity achieved by cancer-related factor were 0.90, 0.89, and 1, respectively. The ROC curve of cancer-related factor is shown in Figure 4A, and the area under the ROC curve (AUC) reached 0.9494. The cancer-related factor constructed by PmBs has high specificity and sensitivity and low computational complexity and does not require training; thus, it has great potential application in auxiliary clinical diagnosis.

**TABLE 3:** Performance of cancer-related factor.

**Actual**			
**Predicted**	**Positive (GA)**	**Negative (control)**	**Total**
True	240	0	240
False	27	12	39
Total	267	12	279

### Sample Classification Power of Classifiers With Machine Learning

The results of fivefold cross-validation of RF-based, SVM-based, and NB-based classifiers using dataset 4 are shown in Table 4. Average accuracy, sensitivity, and specificity of the RF-based, SVM-based, and NB-based classifiers were 0.94, 0.98, and 0.96, 0.94, 0.97, and 0.94, and 1, 1, and 0.97, respectively. The average ROC curves of the three classifiers are shown in Figure 4B, and all three AUCs were above 0.99. This finding provides further proof that PmBs can be potential markers related to GA.

**TABLE 4:** Results of fivefold cross-validation of three classifiers with machine learning.

	**Model**	**First (%)**	**Second (%)**	**Third (%)**	**Fourth (%)**	**Fifth (%)**	**Average (%)**
Accuracy	RF	90.910	92.308	100	88.889	100	94.4211
	SVM	90.909	100	100	100	100	98.1818
	NB	90.909	100	100	88.889	100	95.9596
Sensitive	RF	88.889	100	100	83.333	100	94.4444
	SVM	88.889	100	100	100	100	97.7778
	NB	88.889	100	100	83.333	100	94.4444
Specificity	RF	100	85.714	100	100	100	97.1429
	SVM	100	100	100	100	100	100
	NB	100	100	100	100	100	100

### Survival-Related PmBs in GA

Fourteen survival-related PmBs (LMNB2, BGN, IRAK1, MFSD12, FKBP10, SOX4, SLC12A7, DNMT1, SLC1A5, TIMP1, ENTPD6, GPX3, HELZ2, and PMEPA1) were identified by univariate Cox regression analysis, and the detailed results are shown in Supplementary Table 3. Multivariate Cox regression analysis identified LMNB2, BGN, MFSD12, and SOX4 (refer to Supplementary Table 4), which can be used to construct a prognostic risk model. The risk score of the *i-th* (*i* = 1, 2,⋅, 269) GA sample was calculated as follows:

$$Riskscore\left(i\right)=-0.5295\times Exp_{L\,M\,N\,B\,2}\left(i\right)+0.2133\times Exp_{B\,G\,N}\left(i\right)-0.6516\times Exp_{M\,F\,S\,D\,12}\left(i\right)+0.2814\times E\,x\,p_{S\,O\,X\,4\,\left(i\right)}.$$

Where Exp_α_(*i*) (α is LMNB2, BGN, MFSD12, or SOX4) is the expression value of a survival-related PmB in the *i-th* GA sample.

**FIGURE 5:**
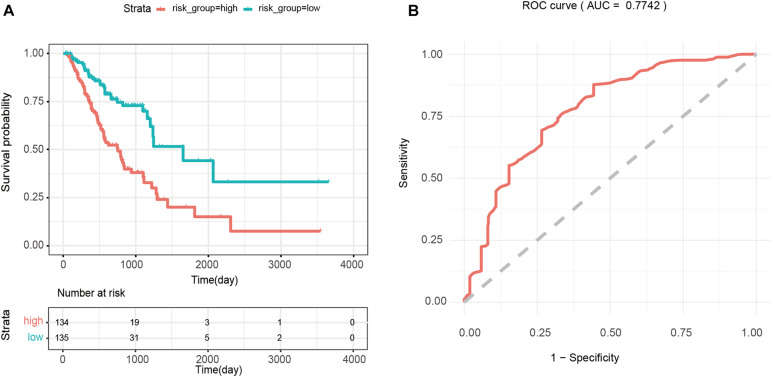
Survival analysis based on four prognostic PmBs. **(A)** Kaplan–Meier curves analysis for overall survival of patients between the high- and low-risk groups; the upper panel represents the Kaplan-Meier curve for the high and low risk groups, the lower panel shows the cumulative number of deaths. **(B)** ROC analysis for prognostic risk model with the 4 PmBs.

The median of the risk scores of all GA samples −(34 in this case) was used as the cutoff value, and 269 patients were divided into the high-risk (>−34, *n* = 134) and low-risk groups (<−8.34, *n* = 135). Kaplan-Meier survival analysis of patients in the high- and low-risk groups showed that the difference between the two groups was significant (*p* < 0.0001). As shown in Figure 5A, the average survival time of patients inhe high-risk group was shorter, and the number of deaths was higher than those of patients in the low-risk group. In addition, the results of ROC analysis showed that the AUC value of the prognostic risk model constructed using 4 PmBs was 0.7742 (Figure 5B), suggesting good specificity and sensitivity.

### Generalization Ability

Generalization ability of mRBioM was assessed on three other complete transcriptomic datasets, including TCGA-COAD (colonic adenocarcinoma), TCGA-LUAD (lung adenocarcinoma), and TCGA-LIHC (hepatocellular carcinoma), downloaded from the TCGA database, and the results are shown in Table 5. Average accuracy and sensitivity of CF were between 0.92 and 0.99, and average specificity was 1. Average accuracy and sensitivity of the RF-based, SVM-based, and NB-based classifiers were between 0.94 and 0.99, and average specificity was above 0.95. Therefore, the classifiers constructed with PmBs have good sample classification power in 3 other cancer datasets, indicating that the mRBioM algorithm has good generalization ability and can effectively identify potential cancer-related mRNA markers in other cancers.

**TABLE 5:** Generalization ability verification results.

**Dataset ID**		**TCGA-COAD**	**TCGA-LUAD**	**TCGA-LIHC**
Disease type		Colon adenocarcinoma	Lung adenocarcinoma	Liver hepatocellular carcinoma
PmBs number		289	200	300
CF	ACC	0.9869	0.9709	0.9384
	SP	1	1	1
	SE	0.9866	0.9688	0.9366
	CF_th_	0.9768	1.001	0.9384
	CF_C_	0.8578–1.1323–1.3027	0.8595–1.2112–1.4593	0.7024–0.9974–1.6711
	CF_N_	0.7603–0.8752–0.9444	0.8159–0.8429–0.9478	0.6199–0.6904–0.8104
RF	ACC	0.9716	0.9846	0.9826
	SP	0.9667	0.9778	0.975
	SE	0.9833	0.975	0.9867
NB	ACC	0.975	0.9833	0.9735
	SP	0.95	1	0.9568
	SE	1	0.9652	0.9833
SVM	ACC	0.9833	0.975	0.9913
	SP	0.9667	1	1
	SE	1	0.9485	0.9833

*ACC, accuracy; SP, specificity; SE, sensitivity; CF_th_, Cancer-related factor value threshold; CF_C_, Cancer-related factor value of cancer sample; CF_N_, Cancer-related factor value of normal sample.*

## Discussion

This study proposed the mRBioM algorithm to identify potential mRNA biomarkers from the complete transcriptomic RNA profiles of GA. Unlike existing algorithms, mRBioM evaluates the potential of each DE mRNA as a biomarker by combining the corresponding amount of information at the transcription and protein levels based on the information entropy theory. Fifty-five DE mRNAs were identified as PmBs associated with GA. These 55 PmBs were used to construct four sample classifiers, including cancer-related factor, RF-based, SVM-based, and NB-based classifiers, to illustrate the reliability of PmBs identified by mRBioM. Good sensitivity, specificity, and accuracy of classification were achieved by the four classifiers. Four of fifty-five PmBs had good ability for prognostic evaluation of the overall survival of GA patients. TCGA-COAD, TCGA-LUAD, and TCGA-LIHC datasets confirmed the generalization ability of mRBioM. The classifiers constructed by the identified PmBs suggested good performance in a variety of classification algorithms and cancer-related datasets, which is expected to be used in more researches on cancer-related biomarker identification.

Thirteen of 55 PmBs (Table 2) were confirmed by the data of the literature to play certain roles in occurrence and development of GA and were biomarkers or potential therapeutic targets of GA. For example, GPRC5A and SOX9 have been shown to be related to occurrence and development of GA (Liu et al., 2016; Wang H. et al., 2020), and their expression levels changed more than fourfold in the GA vs. adjacent control samples according to the result of DE RNA analysis (log_2_FC > 2). FCMET has been confirmed as a resistance factor in GA (Ebert et al., 2019), and Wang R. G. et al. (2020) demonstrated that FKBP10 may be a crucial player mediating cell proliferation, invasion, and migration by regulating the PI3K signaling pathway in GA. Twenty-seven of 55 PmBs (Table 2) were shown to be associated with other cancers according to the data of the literature. Thus, mRBioM identified some new GA-related mRNAs. We attempted to extract additional DE mRNAs as PmBs related to GA. However, adding PmBs did not improve the classification powers of the four classifiers, and these extra PmBs were not associated with prognosis. Thus, our strategy for PmBs screening according to the change trend of the total information amount for all PmBs was effective.

Notably, the value ranges of the cancer-related factor calculated in most cancer and adjacent normal samples of four cancer-related datasets were 0.9–1.4 and 0.7–0.9, respectively. Additionally, the thresholds of cancer-related factors (CF_th_) in all four datasets were approximately 1. The values of cancer-related factors and their corresponding thresholds showed good consistency and robustness in all four datasets. Although the classification performance of cancer-related factor is slightly worse than that of three classifiers with machine learning, the approach does not require training and has considerably lower computational complexity than that of three classifiers with machine learning. Importantly, the method requires only a small number of cancer and adjacent samples to determine the threshold and evaluates whether a single sample corresponds to cancer. Thus, the cancer-related factor may have good application prospects in the personalized diagnosis of cancers.

LMNB2, BGN, MFSD12, and SOX4 in 55 GA-related PmBs were identified and combined into a prognostic risk scoring model. There is no experimental evidence that LMNB2, BGN, and MFSD12 in this combination are associated with GA, and these are new PmBs identified in this study. LMNB2 belongs to the lamin family and is closely related to occurrence, development, and prognosis of liver cancer (Kong et al., 2020; Li X. N. et al., 2020). BGN is an important member of the leucine-rich small proteoglycan family and an important component of the extracellular matrix. Clinical studies have shown that upregulation of BGN is related to poor prognosis of patients with various types of cancer syndromes (Zhao S. F. et al., 2020). MFSD12, also known as PP3501, is a nuclear protein (Wang et al., 2012). Bioinformatic analysis revealed that upregulated expression of MFSD12 is a key promoter of cell proliferation, potential prognostic biomarker, and therapeutic target for melanoma (Wei et al., 2019). SOX4 is a key transcription factor involved in occurrence and development of many cancers (Liu et al., 2018; Wang et al., 2018; Ding et al., 2019) and was shown to be related to the proliferation, migration, and invasion of GA cells and prognosis of GA patients (Fang et al., 2012; Dong et al., 2018; Shao et al., 2020). Therefore, the model has good sensitivity and specificity (AUC = 0.7742), and the risk score calculated by the model can effectively predict the risk of GA patients (*p* < 0.0001, hazard ratio = 2.845, 95% CI: 2.033–3.981).

In conclusion, our study proposes an mRBioM algorithm to identify PmBs from the complete transcriptomic RNA profiles of GA by integrating and analyzing the information at transcriptome and proteome levels. mRBioM identified 55 PmBs related to the occurrence, development and prognosis of GA, which may provide potential biomarkers for early diagnosis, treatment, and prognosis of GA. mRBioM can also be applied in other cancers for cancer-related biomarker identification. But this study also has several limitations. mRBioM is a computational method, and reliability of GA-related PmBs identified by mRBioM was confirmed only by computational methods; thus, further experimental studies are needed to verify the clinical value of identified GA-related PmBs.

## Data Availability Statement

The original contributions presented in the study are included in the article/Supplementary Material, further inquiries can be directed to the corresponding author/s.

## Ethics Statement

Ethical review and approval was not required for the study on human participants in accordance with the local legislation and institutional requirements. The patients/participants provided their written informed consent to participate in this study.

## Author Contributions

CD and NR designed the study and wrote the manuscript. CD, FG, and XL conducted the computer experiments. NR, WD, GW, and JZ analyzed the results and revised and offered advice about the manuscript. All authors participated in the critical review, revision of this manuscript, contributed to the article, and approved the submitted version.

## Conflict of Interest

The authors declare that the research was conducted in the absence of any commercial or financial relationships that could be construed as a potential conflict of interest.

## Publisher’s Note

All claims expressed in this article are solely those of the authors and do not necessarily represent those of their affiliated organizations, or those of the publisher, the editors and the reviewers. Any product that may be evaluated in this article, or claim that may be made by its manufacturer, is not guaranteed or endorsed by the publisher.

## Footnotes

1
http://string-db.org/

## Abbreviations

mRBioM: mRNA Biomarkers
GA: gastric adenocarcinoma
PmBs: potential mRNA biomarkers
DE: differentially expressed
FC: fold change
GO: Gene Ontology
KEGG: Kyoto Encyclopedia of Genes and Genomes
RF: random forest
SVM: support vector machine
NB: naive Bayes
AUC: area under the ROC curve.
